# Segmented fluorescence correlation spectroscopy (FCS) on a commercial laser scanning microscope

**DOI:** 10.1038/s41598-024-68317-7

**Published:** 2024-07-30

**Authors:** Elisa Longo, Silvia Scalisi, Luca Lanzanò

**Affiliations:** 1 Department of Physics and Astronomy “Ettore Majorana”, University of Catania, Via S. Sofia, 64, 95123 Catania, Italy; 2 Nanoscopy, CHT Erzelli, Istituto Italiano di Tecnologia, Genoa, Italy

**Keywords:** Fluorescence correlation spectroscopy (FCS), Confocal laser scanning microscope (CLSM), Segmented FCS, Diffusion coefficient, GFP, PARP1, Biophysics, Biological fluorescence, Confocal microscopy

## Abstract

Performing accurate Fluorescence Correlation Spectroscopy (FCS) measurements in cells can be challenging due to cellular motion or other intracellular processes. In this respect, it has recently been shown that analysis of FCS data in short temporal segments (segmented FCS) can be very useful to increase the accuracy of FCS measurements inside cells. Here, we demonstrate that segmented FCS can be performed on a commercial laser scanning microscope (LSM), even in the absence of the dedicated FCS module. We show how data can be acquired on a Leica SP8 confocal microscope and then exported and processed with a custom software in MATLAB. The software performs segmentation of the data to extract an average ACF and measure the diffusion coefficient in specific subcellular regions. First of all, we measure the diffusion of fluorophores of different size in solution, to show that good-quality ACFs can be obtained in a commercial LSM. Next, we validate the method by measuring the diffusion coefficient of GFP in the nucleus of HeLa cells, exploiting variations of the intensity to distinguish between nucleoplasm and nucleolus. As expected, the measured diffusion coefficient of GFP is slower in the nucleolus relative to nucleoplasm. Finally, we apply the method to HeLa cells expressing a PARP1 chromobody to measure the diffusion coefficient of PARP1 in different subcellular regions. We find that PARP1 diffusion is slower in the nucleolus compared to the nucleoplasm.

## Introduction

Fluorescence Correlation Spectroscopy (FCS) is a well-established experimental method to measure the diffusion coefficient of fluorescent molecules or particles^1,2^. FCS finds application in several fields, including biochemistry, biophysics, molecular biology, and material sciences. FCS analyzes the spontaneous fluctuations of fluorescence intensity arising from fluctuations in the concentration of particles in a given observation volume. The fluctuations are characterized through an autocorrelation function (ACF) whose width depends on the diffusion coefficient of the particles and whose amplitude depends on the number of particles N in the observation volume. For reasons related to the increase of the signal-to-background ratio, FCS is typically performed in small observation volumes and with small values of *N*: for instance, a concentration of 0.1 μM and a femtoliter (1 fL = 10^–15^ L) observation volume correspond to N ≈ 60 particles. Even if its original implementation was based on a small volume generated in a cuvette, today the most common FCS setup is based on a confocal microscope equipped with a high numerical aperture (NA) objective ^3^.

Several FCS techniques have stemmed from the basic single-point architecture. Techniques that parallelize the FCS acquisition, based on the use of fast cameras^4–7^ or detector arrays ^8–10^, enable the simultaneous detection of fluctuations at multiple sample locations. Spot variation techniques, based on the modulation of optical elements^11,12^, the combination with stimulated emission depletion (STED)^13,14^ or the use of confocal detector-arrays^15,16^ enable the measurement of diffusion properties at different spatial scales. In scanning FCS techniques, fast line or circular scanning can be used to collect data at multiple locations, generate diffusion maps and/or analyze the cross-correlation between different pixels^17–22^.

Another class of methods has extended FCS analysis to images acquired on commercially available laser scanning microscopes (LSM), promoting the use of FCS also among non-expert users. In these methods, data are acquired on a commercial LSM setup and analyzed using dedicated software. Raster image correlation spectroscopy (RICS)^1^ and subsequent variants^15,23,24^ exploit the spatial and temporal information encoded in the raster-scan pattern of a LSM to measure diffusion coefficients of molecules in solutions and cells. Similarly, STICS^25^ and iMSD^26^ exploit the analysis of spatiotemporal correlations between different pixels of a time-lapse acquisition to detect different types of motion, including deviations from Brownian diffusion. In all these approaches, the acquired images are processed with algorithms that analyze the correlation in space and time and extract the diffusion coefficient and/or other relevant parameters.

Recently^27^, Di Bona et al have shown that, by slowly and continuously scanning the focal spot across the sample, one could acquire FCS data on multiple locations inside the cell nucleus with high accuracy^27^^.^ The continuous dataset was divided into short temporal segments and the fluctuation analysis was performed by considering each segment as an independent FCS measurement that generates its own ACF. The ACFs calculated from these short segments were sorted into two or more populations (e.g. corresponding to different nucleus regions) and then averaged. As a result, this segmented FCS approach yielded, with a single measurement, average ACFs corresponding to different chromatin regions, enabling, for instance, accurate comparison of mobility of GFP in euchromatin versus heterochromatin or the characterization of the mobility of a receptor close to a gene array^27^. The effect of segmentation on FCS data has been thoroughly investigated by Kohler et al. ^28^. In particular, if the segment size is too short, the procedure for fitting the ACF must be modified^28^.

Here, we show how segmented FCS data can be extracted in a commercial laser scanning microscope, even in the absence of a dedicated FCS module. Data can be acquired on a Leica SP8 confocal microscope and then exported and processed with a custom script in MATLAB to generate the corresponding ACFs. The acquired data are first divided into segments, and each segment generates an ACF. The segments are then sorted and selected to generate average ACFs corresponding to specific regions of the sample. The average ACFs are analyzed in the same way as in single-point FCS. In this way, we obtain the diffusion coefficient (or other FCS-related parameters) in different specimen zones. We describe two types of LSM data acquisition and segmentation: (i) X-segmentation, where fluctuations are analyzed between consecutive pixels, provides segmented FCS data with the maximum temporal resolution and can be used to measure the fast diffusion of fluorophores in solutions or cells; (ii) Y-segmentation, where fluctuations are analyzed between pixels of consecutive lines, is more suitable for measuring slower dynamics like those of molecules interacting inside cells.

First, we show that good-quality ACFs can be obtained in a commercial LSM using our method. To this aim, we measure the diffusion of fluorophores of different sizes (from about 1 nm to 100 nm) in solution. Then, we validate the segmentation approach by measuring the diffusion coefficient of untagged GFP in the nucleus of HeLa cells, exploiting intensity variations to distinguish between nucleoplasm and nucleolus. As expected, the measured diffusion coefficient of GFP is slower in the nucleolus (D_nucleolus_ = 9.5 ± 3 µm^2^/s) relative to nucleoplasm (D_nucleoplasm_ = 29 ± 4 µm^2^/s) due to the higher level of molecular crowding of the nucleolar compartment and in keeping with previous reports. Finally, we apply the method to HeLa cells expressing a PARP1 chromobody tagged with RFP (PARP1-chr-RFP) to measure the diffusion coefficient of PARP1 in different subcellular regions. We estimate a diffusion coefficient for PARP1 in the nucleoplasm D_nucleoplasm_ = 1.3 ± 0.4 µm^2^/s, as expected for a protein which interacts with chromatin. Notably, we find that the diffusion coefficient of PARP1 is slower in the nucleolus (D_nucleolus_ = 0.4 ± 0.2 µm^2^/s), probably due to a higher level of binding in this compartment.

## Theory

### Extraction of segmented FCS data in a laser scanning microscope

**Figure 1:**
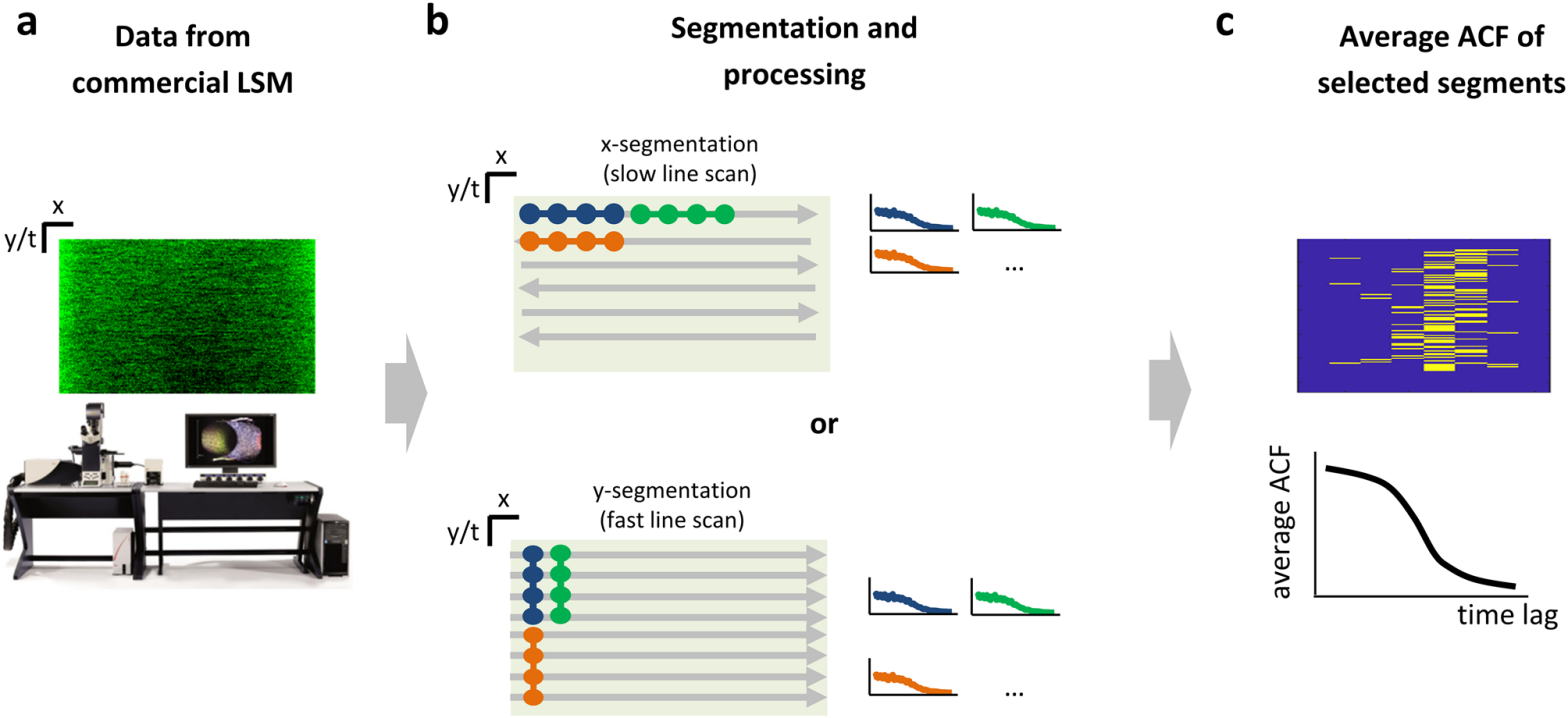
Extraction of segmented FCS data on a commercial microscope. (**a**) Raw data are acquired from a commercial laser scanning microscope and exported as images. (**b**) Schematic of the data segmentation and processing procedure: (top) for X-segmentation, data acquired by slow scanning of the X-axis are segmented along the X direction; (bottom) for Y-segmentation, data acquired by fast scanning along the X-axis are segmented along the Y direction. For each segment, the corresponding autocorrelation function (ACF) is generated. (**c**) The average intensity value calculated in each segment is shown on a map and used to select the segments corresponding to a specific region of the sample. The final ACF is obtained as the average of the ACFs of the selected segments and exported for further analysis.

Here, we discuss how to extract segmented FCS data from a laser scanning microscope (LSM) (Fig. 1a–c). Specifically, we describe how to segment and analyze fluctuations along a single axis (X or Y) of the acquired image.

The intensity autocorrelation function (ACF) of a line along direction k (where k is X or Y) is given by:

1 $$G\left(\xi \right)=\frac{\langle I\left(k\right)I\left(k+\xi \right)\rangle }{{\langle I\left(k\right)\rangle }^{2}}-1$$

where I(k) is the fluorescence intensity at pixel position k, ξ represents the spatial lag in pixels, and the brackets indicate averaging over all the pixels of the line. Since the scanned line I(k) contains spatial and temporal information related to the diffusion of the probe and the microscope scanning speed, the spatial correlation of the line G(ξ) also contains spatial and temporal information. The ACF is then fitted to a proper diffusion model to extract the relevant parameters.

The ACF shape depends on two contributions:

2 $$G\left(\xi \right)={G}_{\text{diff}}\left(\xi \right)\cdot S\left(\xi \right)$$

where *G*_diff_(ξ) is the component related to diffusion and *S*(ξ) is the spatial component of the correlation function related to the laser scanning. For a Gaussian 2D diffusion model:

3 $$G\left(\xi \right)={G}_{\text{diff}}\left(\xi \right)\cdot S\left(\xi \right)=G\left(0\right)\cdot \frac{1}{1+\frac{4D|\xi |\delta t}{{w}_{0}^{2}}}\cdot \text{exp}\left(-\frac{{\left(\frac{|\xi |\delta k}{{w}_{0}}\right)}^{2}}{1+\frac{4D|\xi |\delta t}{{w}_{0}^{2}}}\right)$$

where δk is the pixel size and δt is the pixel time (for k = X) or the line time (for k = Y), G(0) is the amplitude of the ACF, D is the diffusion coefficient and w_0_ is 1/e^2^ size of the focal spot in the lateral direction. 

Given that τ = ξδt and that the scanning speed along k is v = δk/δt, Eq. (3) can be converted into a temporal autocorrelation function:

4 $$G\left(\uptau \right)={G}_{\text{diff}}\left(\uptau \right)\cdot S\left(\uptau \right)=G\left(0\right)\cdot \frac{1}{1+\frac{4D\uptau }{{w}_{0}^{2}}}\cdot \text{exp}\left(-\frac{{\left(\frac{v\uptau }{{w}_{0}}\right)}^{2}}{1+\frac{4D\uptau }{{w}_{0}^{2}}}\right)$$

When the scanning speed is low, the component due to scanning is negligible and the ACF is identical to a single-point ACF^27^:

5 $$G\left(\uptau \right)={G}_{\text{diff}}\left(\uptau \right)=G\left(0\right)\cdot \frac{1}{1+\frac{4D\uptau }{{w}_{0}^{2}}}$$

Segmentation of the FCS data is obtained by restricting averaging in Eq. (1) to temporal segments of duration T:

6 $${G}_{j}\left(\xi \right)=\frac{{\langle I\left(k\right)I\left(k+\xi \right)\rangle }_{T}}{{{\langle I\left(k\right)\rangle }_{T}}^{2}}-1$$

As a result, we get as many ACFs G_j_(ξ) as the temporal segments. For data acquired on a LSM, the duration of the segment can also be written as T = n_seg_δt, where n_seg_ is the number of pixels in a segment. The segments can then be sorted (for instance, based on the average value of fluorescence intensity in the segment) and selected to generate an average ACF, calculated as:

7 $${G}_{av}\left(\uptau \right)=\langle {G}_{j}\left(\uptau \right)\rangle $$

where the brackets indicate averaging over all selected segments.

We consider two practical implementations for generating segmented FCS data in an LSM: (i) X-segmentation; (ii) Y-segmentation.

For X-segmentation (Fig. 1b, top), we acquire data by slow scanning the X-axis, with analysis of fluctuations between pixels of the same line. In this case, the temporal resolution is maximum (≈µs) and can be used to measure the fast diffusion of fluorophores in solutions. An advantage is that data acquired in ‘bidirectional’ scan mode (i.e. when data are also recorded during retracing) can be analyzed in the same way as data acquired in monodirectional scan mode. The main limitation is that the maximum segment duration, T, corresponds to the duration of a line, T ≤ T_line_. The scanned line is characterized by a length in pixels N_px_ and thus by a corresponding duration T_line_ = N_px_δt and length L_line_ = N_px_δx. The duration T sets a physical limit for the observation of fluctuations. As discussed previously, the thumb rule is that T must be at least two orders of magnitude longer than the characteristic correlation time^19,27^ unless data are analyzed with a special approach ^28^. In our Leica SP8 microscope, when we set the minimum value of line frequency available in the setup (f = 1 Hz) and N_px_ = 8192 pixels, we find δt = 30.5 µs and thus T_line_ = 0.25 s. If we set the line frequency to 10 Hz, we get a better temporal resolution, δt = 3.05 µs, but a shorter line duration, T_line_ = 25 ms. The length, L_line_, determines the region of the sample explored during the measurement. In our Leica SP8 microscope, when we set the maximum zoom, we find δx = 0.47 nm and thus L_line_ = 3.84 µm. The chosen values of pixel size and pixel time also determine the scanning speed v (v = 154 µm/s at 10 Hz, v = 15.4 µm/s at 1 Hz).

For Y-segmentation (Fig. 1b, bottom), we acquire data in the scanning-FCS mode, i.e. by fast line scanning along the X-axis. The analysis of fluctuations is performed between pixels of different lines. Thus, the temporal resolution is given by the line time, i.e. the inverse of the line scanning frequency, typically in the order of ~ 1 ms (~ 0.1 ms for resonant scanners). In this case, there is no limit to the segment duration T, as it depends only on the length of the acquisition, T ≤ T_exp_, with T_exp_ = N_lines_δt, where N_lines_ is the total number of lines acquired and δt is the line time. For instance, by acquiring N_lines_ = 200,000 lines at a line frequency of 1800 Hz, we get a line time δt = 0.55 ms and T_exp_ = 110 s.

## Materials and methods

### Preparation of fluorescent probe solutions

Solutions of different-sized fluorophores were prepared to test the goodness of our methods. Purified GFP (rAcGFP1 Protein, Clontech, 632502) was diluted in phosphate-buffered saline (PBS) to a final concentration of 0.01 mg/ml and deposited on a 3% BSA-coated chambered coverslip (Ibidi, µ-slide 8 well glass bottom, 80821). Alexa Fluor 488 azide (Invitrogen, A10266) was diluted in PBS to a final concentration of ~ 100 nM. A goat anti-mouse IgG secondary antibody conjugated to the Alexa Fluor 488 (Abcam, AB150113) was used at the concentration of 2 µl/ml in PBS. Yellow-green fluorescent spheres with a size of 100 nm (Thermo Fisher Scientific, F8803) were sonicated and diluted in PBS.

### Cell culture and transfection

HeLa cells (ATCC n. CCL-2™) were cultured in DMEM (Dulbecco’s modified Eagle’s medium, Gibco™, 11965092) supplemented with 10% fetal bovine serum (Euroclone, ECS5000LH) and 1% penicillin/streptomycin (Life Technologies, 15140-122).

For fluorescence microscopy measurements, 20,000 cells/cm^2^ were seeded on eight-well Ibidi chambered coverslips and incubated at 37 °C in 5% CO_2_ for 24 h.

For untagged GFP and PARP1 (poly-[ADP-ribose] polymerase 1) diffusion measurements, cells were transiently transfected using Lipofectamine 3000^®^ transfection kit (Thermo Fisher Scientific, L3000-001) with an untagged GFP-containing plasmid, or PARP1-chromobody-TagRFP plasmid (ChromoTek, xcr), respectively. After transfection cells were incubated at 37°C in 5% CO_2_ for 24 h.

### Data acquisition

All measurements were performed on a Leica TCS SP8 confocal laser scanning microscope, using a 1.40 NA 63× objective (HCX PL APO CS2 63/1.40 Oil Leica Microsystems).

For GFP, Alexa 488 azide and IgG secondary antibody acquisition we used an excitation wavelength of 488 nm, with emission detection bands 500–530 nm and 535–600 nm via hybrid detectors operating in photon counting mode. For PARP1-chromobody-TagRFP (PARP1-Chr-RFP) measurements, the excitation wavelength was set at 561 nm, with emission detection bands 570–600 nm and 605–650 nm via hybrid detectors operating in photon counting mode. We used detection in two channels to cross-correlate the signal and remove the detector noise.

For all the fluorophores in solution, data were acquired as XY (raster scan) images in bidirectional slow scanning mode by setting the scanner velocity at a line frequency of 10 Hz, corresponding to 154 µm/s, with a temporal resolution of 3.04 µs.

For untagged GFP in cells, data were acquired as XT (line scan) images in bidirectional slow scanning mode by setting the scanner velocity at a line frequency of 1 Hz (which is the minimum value allowed by the acquisition software), corresponding to 15.4 µm/s, with a temporal resolution of 30.4 µs. The final image format was 8192 × 128 pixels and a pixel size of 0.47 nm.

For PARP1-Chr-RFP in cells, data were acquired as XT (line scan) images in fast scanning mode by setting the scanner velocity at a line frequency of 1800 Hz (corresponding line time 0.56 ms), obtaining an image format of 128 × 204,800 pixels and a pixel size of 194 nm.

### Data processing and analysis

The line scan (XT) or raster scan (XY) data, saved as .lif files, are opened in ImageJ and then saved as TIF files. The TIF files are opened with a custom script in MATLAB (Mathworks) and processed for segmentation and calculation of the autocorrelation function (ACF). Each file is an image with dimensions N_px_ × N_lines_. For X-segmentation, the data are segmented along X with a segment length set to n_seg_ ≤ N_px_, resulting in a total number of segments ~ (N_px_/n_seg_)N_lines_. For Y-segmentation, the data are segmented in Y with a segment length set to n_seg_ ≤ N_lines_, resulting in a total number of segments ~ (N_lines_/n_seg_)N_px_. For each segment, the software calculates the spatial ACF given by (Eq. 1), where the numerator is calculated using a Fourier Transform algorithm. For data acquired splitting the signal in two channels, the ACF is calculated as the cross-correlation of the two channels. The spatial ACF is converted into a temporal ACF using the value of pixel time (X-segmentation) or line time (Y-segmentation) used for acquiring the data. The pixel time was provided by the Leica SP8 software. The line time was calculated as 1/f, where f is the line scanning frequency set in the Leica SP8 software. The average intensity value calculated in each segment is shown on a map and used to select the segments. The segment intensity value is normalized to the maximum value of each X line to correct for variations along Y or T (e.g. due to photobleaching) and highlights only relative intensity variations along X. The final ACF is calculated as the average of the ACFs of the selected segments and exported for further analysis. The software is available as source code (requiring MATLAB) or a standalone distribution for Windows at https://github.com/llanzano/SegmentedFCS.

The ACF were fitted in Origin (OriginLab, Northampton, MA, USA) using one of the following models, corresponding to diffusion only, diffusion with scanning, diffusion with scanning and triplet dynamics, respectively:

8 $$G\left(\uptau \right)={G}_{\text{diff}}\left(\uptau \right)=G\left(0\right)\cdot \frac{1}{1+\frac{4D\uptau }{{w}_{0}^{2}}}$$

9 $$G\left(\uptau \right)={G}_{\text{diff}}\left(\uptau \right)\cdot S\left(\uptau \right)=G\left(0\right)\cdot \frac{1}{1+\frac{4D\uptau }{{w}_{0}^{2}}}\cdot \text{exp}\left(-\frac{{\left(\frac{v\uptau }{{w}_{0}}\right)}^{2}}{1+\frac{4D\uptau }{{w}_{0}^{2}}}\right)$$

10 $$G\left(\uptau \right)={G}_{\text{diff}}\left(\uptau \right)\cdot S\left(\uptau \right)\cdot T\left(\uptau \right)=G\left(0\right)\cdot \frac{1}{1+\frac{4D\uptau }{{w}_{0}^{2}}}\cdot \text{exp}\left(-\frac{{\left(\frac{v\uptau }{{w}_{0}}\right)}^{2}}{1+\frac{4D\uptau }{{w}_{0}^{2}}}\right)\left(1+{\text{A}}_{tr}\cdot \text{exp}\left(-\frac{\uptau }{{\uptau }_{tr}}\right)\right)$$

where G(0) is the amplitude of the ACF, where D is the diffusion coefficient and w_0_ is 1/e^2^ size of the focal spot in the lateral direction, v is the speed of the scanner, A_tr_ = f_tr_/(1-f_tr_) where f_tr_ is the triplet fraction, τ_tr_ is the triplet time constant. Equation (8) was used to fit data acquired in fast line scan (XT) mode and processed by Y-segmentation (since the speed along Y is zero). Equation (9) was used to fit data acquired in slow line scan (XT) mode and processed by X-segmentation (to take into account the speed of the scanner along X). Equation (10) was used to fit data acquired in slow line scan (XT) in the presence of a triplet component.

The hydrodynamic radius R_H_ corresponding to a measured diffusion coefficient was estimated using the Stokes–Einstein relationship:

11 $$D=\frac{{k}_{B}{T}_{exp}}{6\pi \eta {R}_{H}}$$

where k_B_ is the Boltzmann constant, T_exp_ = 20 C is the temperature of the solution, η = 0.001 Pa⋅s is the viscosity of water.

## Results

### Validation of the method with fluorescent probes in solution

**Figure 2:**
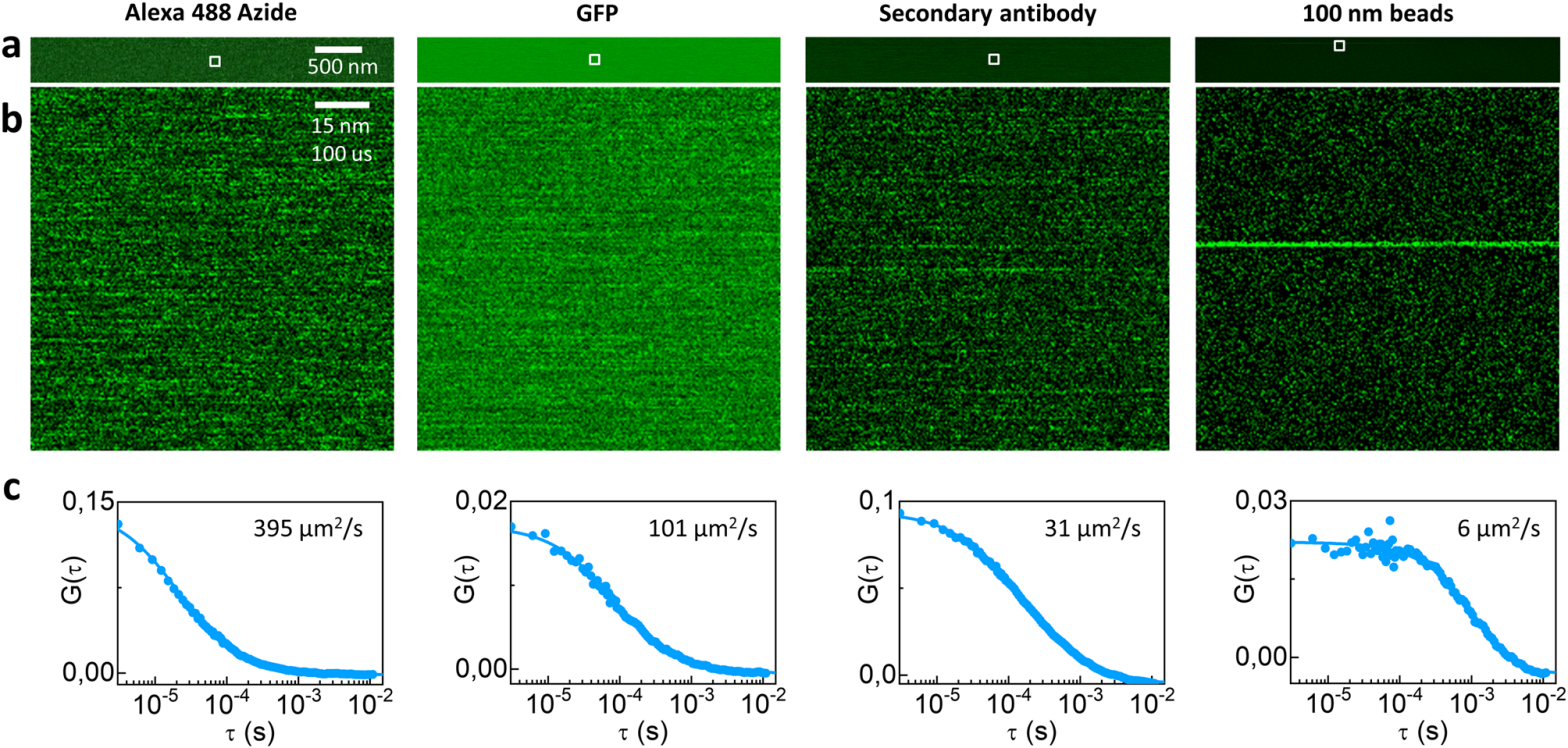
Validation of the method with solutions of different-sized probes (X segmentation). (**a**) Representative data from fluorescent solution acquired by slow scanning along the X axis. (**b**) Zoom-in view in the region indicated by the white square. Note that the temporal fluctuations are visible only along the X-axis of the images. (**c**) Average ACF calculated by averaging the ACFs generated by each X line. By fitting the average ACF to a model of diffusion [Alexa 488 Azide, Eq. (9); GFP, Eq. (9); secondary antibody, Eq. (10); beads, Eq. (9)] we extract the diffusion coefficient values for the different fluorophores.

As a preliminary step, we tested if good-quality ACFs could be obtained from data acquired on the commercial LSM available in our facility, namely the Leica SP8 confocal. To this aim, we measured the diffusion of fluorophores of different sizes (from about 1 nm to 100 nm) in aqueous solution, at room temperature. We acquired raster-scan images characterized by slow bi-directional scanning along the X axis (Fig. 2a). The line frequency was set to 10 Hz, the pixel size was 0.47 nm, the pixel time was δt = 3.05µs. In these conditions, the temporal fluctuations are visible along the X axis of the images (Fig. 2b). Each X line of the image was used as a segment (T = 25ms) for calculating an ACF, and the average ACF of all the segments was reported (Fig. 2c).

The fastest molecule we tested was Alexa 488 Azide (MW = 861 Da). This was also used for calibration of the lateral size w of the observation volume. The ACFs were fitted to a model of diffusion with scanning (Eq. 9). Assuming for Alexa 488 Azide a diffusion constant D = 395 µm^2^/s (calculated from the reported value in Ref.^29^, taking into account the difference in molecular weight), the lateral size was determined to be w = 0.181 ± 0.003 µm. For GFP in solution, the ACFs were also fitted to a model of diffusion with scanning (Eq. 9) and we found D = 106.4 ± 1.5 µm^2^/s (mean ± s.d., n = 3). According to the Stokes–Einstein relationship, this D value corresponds to a hydrodynamic radius R_GFP_ ~ 2 nm, compatible with the molecular weight of GFP (26.9 kDa), and is in keeping with previously reported values^14^. Then we measured the diffusion of a secondary antibody labeled with Alexa 488. In this case, the ACFs were fitted to a model of diffusion with scanning and triplet (Eq. 9) to take into account the triplet component and we found D = 48.6 ± 1.2 µm^2^/s (mean ± s.d., n = 3). According to the Stokes–Einstein relationship, this value corresponds to a hydrodynamic radius R_antibody_ ~ 4 nm, compatible with an estimated molecular weight of ~ 150kDa, and is in keeping with previous reports^15^. Finally, we measured the diffusion of 100-nm diameter fluorescent spheres. In this case, the ACFs were fitted to a model of diffusion with scanning (Eq. 9) and we found D = 6.6 ± 1.5 µm^2^/s (mean ± s.d., n = 4). According to the Stokes–Einstein relationship, this value corresponds to a hydrodynamic radius R_spheres_ ~ 32 nm or a diameter of 64 nm, which is smaller than the nominal size of the fluorescent spheres. However, for a more accurate comparison, the actual size of nanoparticles should be determined by electron microscopy analysis.

### Segmented FCS of untagged GFP in live cells

**Figure 3:**
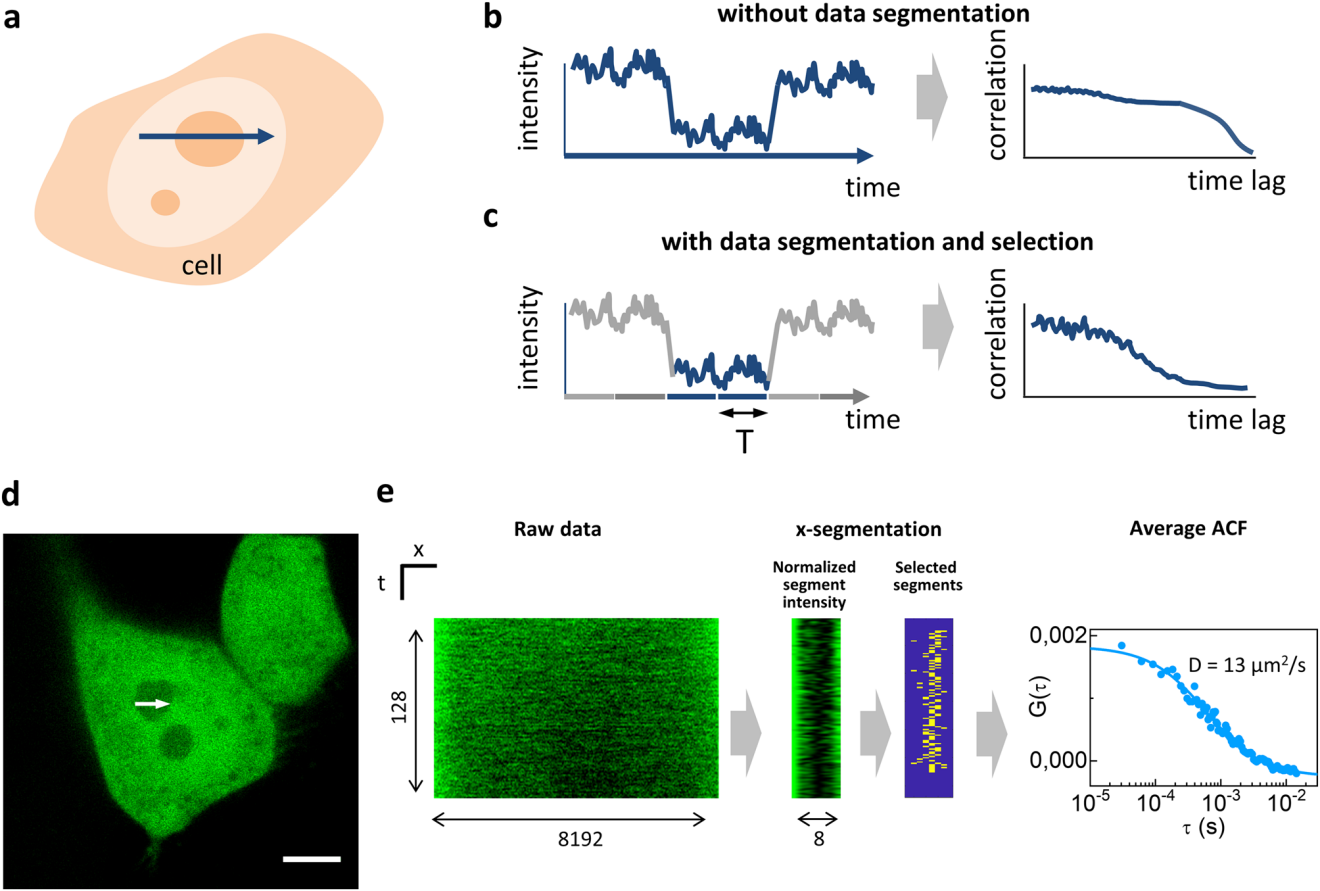
Segmented slow scan FCS of GFP in live HeLa cells. (**a**) Schematic of the slow line scan inside a cell. At the maximum zoom available, the scanner passes across various subcellular regions, such as the nucleolus and nucleoplasm, leading to large intensity variations that deform the autocorrelation function (ACF) (**b**). Data segmentation enables the ACF calculation in short segments of duration T, avoiding the ACF deformations and providing the extraction of ACF in a specific zone (**c**). (**d**) Representative image of a HeLa cells expressing untagged GFP, where the nucleolus can be easily identified as the region with lower GFP intensity. The white arrow indicates the slow line scan region. Scale bar 5 µm. (**e**) Example of data acquisition and processing (from left to right): the raw data is a slow line scan acquisition (XT); X-segmentation is performed dividing each line into 8 segments and each segment is processed to generate an ACF; the segments are selected on the segment intensity map; the average ACFs of the selected segments is obtained and analyzed in the same way as in single-point FCS, to extract the diffusion coefficient.

Next, to demonstrate applicability of the X-segmentation method in live cells, we measured diffusion of untagged GFP in live HeLa cells. To this aim, we performed slow line scanning across the nucleus of HeLa cells expressing GFP (shown schematically in Fig. 3a–c). At the maximum zoom available, corresponding to 3.84 µm, the scanner crosses different subcellular regions (e.g. nucleolus and nucleoplasm) producing large variations of intensity that deform the ACF (Fig. 3b). Segmentation of the data restricts calculation of the ACF to time interval T and enables the extraction of ACF in a specific zone (Fig. 3c).

A representative image of a HeLa cell expressing untagged GFP is reported on Fig. 3d. The nucleolus can be easily identified as the nuclear region with lower GFP intensity. A representative dataset is reported on Fig. 3e. Data are obtained at the minimum frequency allowed by the scan (Line frequency 1 Hz). The GFP intensity along the scan is lower in the nucleolus compared to nucleoplasm. Using X-segmentation, each 8192-pixels line is divided into 8 segments of 1024 pixels. The average intensity of each segment (normalized to the maximum of each row) is used to select the segments corresponding to the nucleolus and calculate the corresponding average ACF (fitted to Eq. 8). Using this approach, we measured the diffusion coefficient of untagged GFP in the nucleoplasm versus the nucleolus. We found D = 29 ± 4 µm^2^/s (mean ± s.d., n = 6) in the nucleoplasm and D = 9.5 ± 3 µm^2^/s (mean ± s.d., n = 9) in the nucleolus. These results are in keeping with previous FCS measurements of untagged GFP in the nucleus ^23,27,30,31^ . Note that we observe some photobleaching of GFP (Supplementary Fig. S1). However, the dynamics of photobleaching does not affect the shape of the ACF because the ACF is calculated in short segments (T = 30ms).

### Segmented FCS of PARP-1 chromobody in live cells

**Figure 4:**
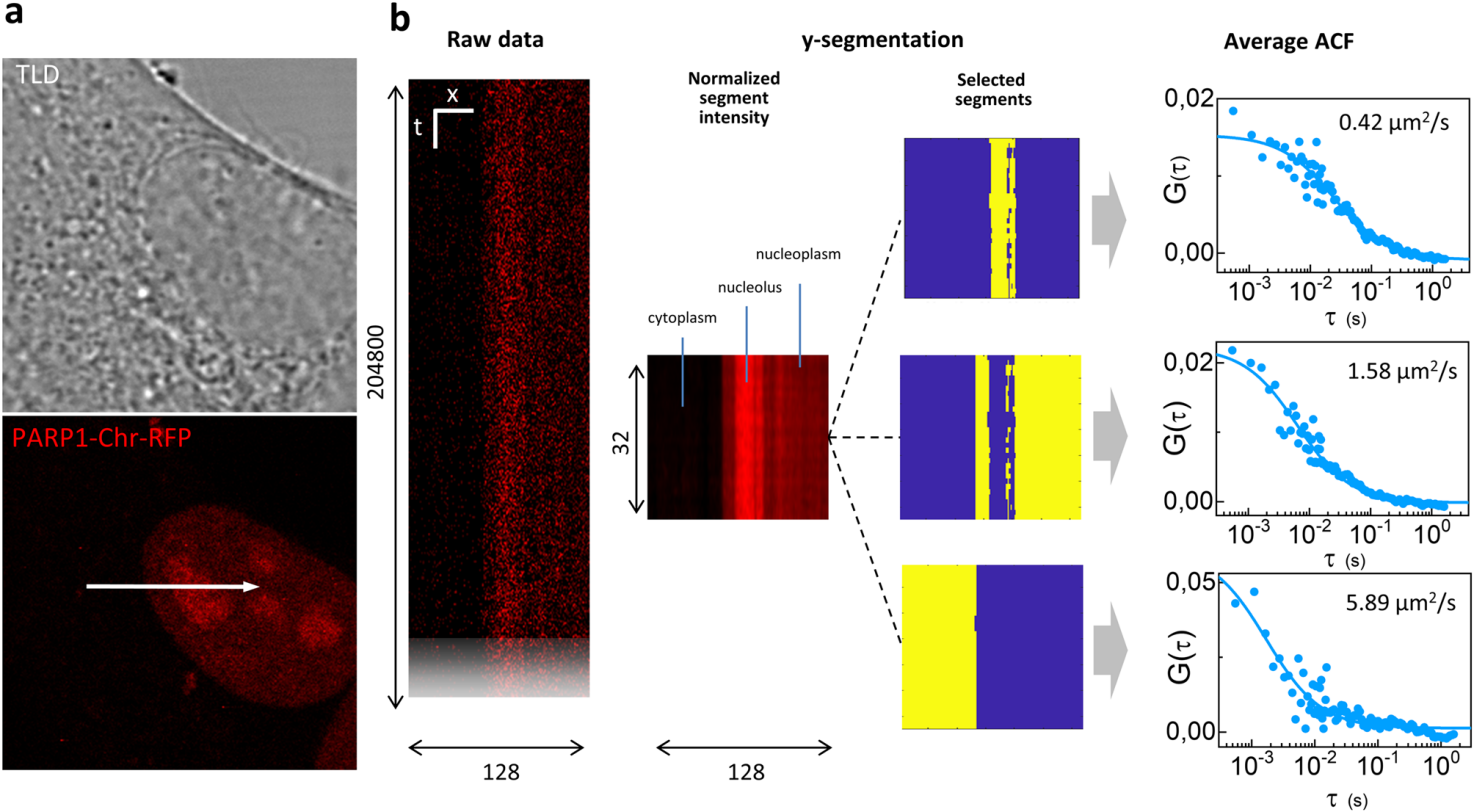
Segmented fast scan FCS of PARP1-chromobody-RFP in live HeLa cells. (**a**) (Bottom) Representative image of a HeLa cells expressing a PARP1 chromobody tagged with RFP, where nucleolus can be easily identified as the region with the highest level of intensity; (top) bright-field image obtained using the transmitted light detector (TLD) of the Leica SP8. (**b**) Example of data acquisition and processing (from left to right): the raw data is a fast line scan acquisition (XT); Y-segmentation is performed dividing each column into 32 segments and each segment is processed to generate an ACF; the segments are selected on the segment intensity map, to identify the nucleolus (high intensity), the nucleoplasm (medium intensity) and the cytoplasm (low intensity); the average ACFs of the selected segments is obtained and fitted to a model of diffusion (Eq. 8) to extract the diffusion coefficient in the three subcellular regions.

Finally, to demonstrate applicability of the Y-segmentation method in live cells, we measured diffusion of a PARP-1 chromobody tagged with RFP (PARP1-Chr-RFP) in live HeLa cells. The PARP-1 chromobody is a small (113 kDa) fluorescent probe that binds with high specificity to the DNA-damage repair protein PARP-1 ^32^. HeLa cells expressing PARP1-Chr-RFP exhibit a clear nuclear signal with an enrichment in the nucleoli (Fig. 4a), as reported previously ^32^. To measure the mobility of PARP1-Chr-RFP we performed fast line scanning (line frequency 1800 Hz) across the cytoplasm and nucleus of transfected cells.

A representative dataset is reported on Fig. 4b. The PARP1-Chr-RFP intensity is higher in the nucleolus compared to nucleoplasm. The cytoplasm exhibits much weaker intensity. Using Y-segmentation, each 204,800-pixels column is divided into 32 segments of 6400 pixels. The average intensity of each segment (normalized to the maximum of each row) is used to select the segments corresponding to specific regions and calculate the corresponding average ACFs (Fig. 4b). All the ACFs were fitted to Eq. (8). Using this approach we found the diffusion coefficient of PARP1 in the nucleoplasm, D = 1.3 ± 0.4 µm^2^/s (mean ± s.d., n = 8). This value is in keeping with previously reported values of mobility for fluorescent labeled PARP-1 in the nucleus^33–35^. In the nucleolus, the mobility of PARP1 was slowed down to D = 0.4 ± 0.2 µm^2^/s (mean ± s.d., n = 8), probably due to an increased level of binding in this region. To the best of our knowledge, this is the first time that the different mobility of PARP1 in the nucleoplasm and in the nucleolus is measured by FCS. In the cytoplasm, where the signal of PARP1-Chr-RFP is very low, we found D = 4.5 ± 2.7 µm^2^/s (mean ± s.d., n = 8). This could be ascribed to a faster diffusion of cytoplasmic PARP-1. Another possible explanation is that this fast component is due to the presence of unbound chromobody. Note that we observe photobleaching of PARP1-Chr-RFP during the acquisition (Supplementary Fig. S2). However, also in this case the dynamics of photobleaching does not affect the shape of the ACF because the ACF is calculated in short segments (T = 3.5s).

## Discussion

We have demonstrated that segmented FCS can be performed on a commercial laser scanning microscope not equipped with a FCS module. In general, while segmentation of the data is not necessary for measurements of fluorescent species in homogeneous solution, it can be very useful to increase the accuracy of FCS measurements inside cells. Indeed, the accuracy of FCS in cells is often hampered by cell and organelle motion, photobleaching or other intracellular processes. Segmentation of FCS data is useful to filter out slower dynamics components and to generate independent segments that can be averaged together to provide an ACF corresponding to a specific subcellular region.

A very popular method for performing fluorescence fluctuation spectroscopy on commercial LSM is Raster Image Correlation Spectroscopy (RICS). In RICS, segmentation of data has been proposed using arbitrary image regions ^24^ or small square regions^15^. Compared to RICS, the main difference is that here we analyze correlations along a single axis (either X or Y/T) and analyze the data in a single-point-like fashion (i.e. we analyze a 1-dimensional ACF whereas in RICS the analysis is done using a 2-dimensional ACF). For this reason, we perform data acquisition either in slow X scanning mode, analyzing correlations only along X, or in fast X scanning mode, analyzing correlations only along Y or T. In both cases, we acquire data with a large number of pixels in the direction of segmentation (e.g. 8192 × 128 for X-segmentation or 128 × 204,800 for Y-segmentation) in such a way that multiple data segments can be obtained from that direction. In our approach, the relevant information is fully contained in a 1-dimensional correlation function (like in single-point FCS), whereas in RICS the information is contained in a 2-dimensional correlation function. The proposed segmented FCS method could be integrated in the future as an additional tool in already available MATLAB based packages dedicated to FCS and RICS^36^.

**Figure 5:**
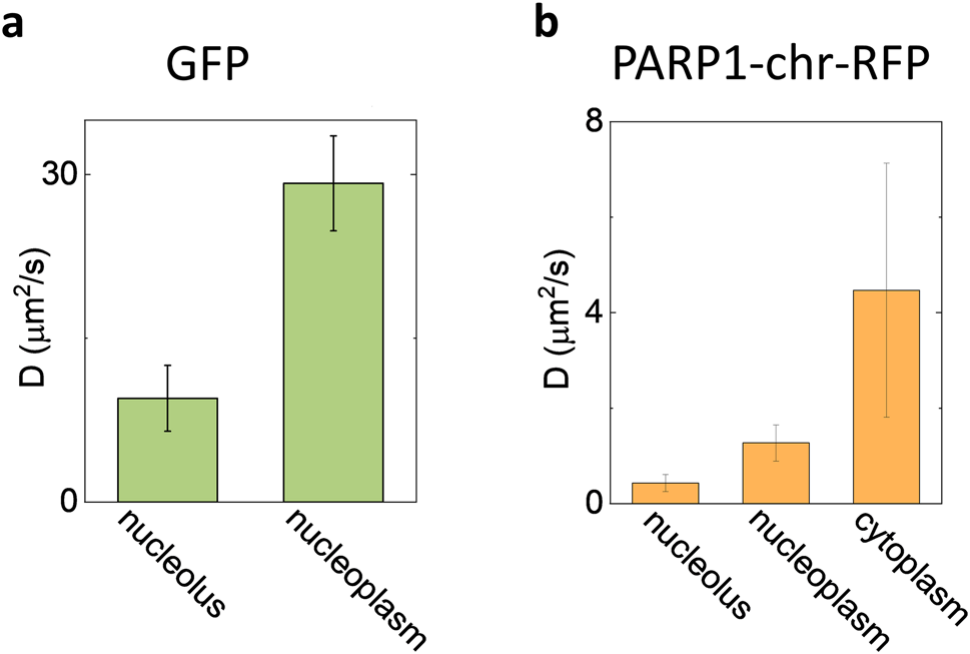
Diffusion coefficients of GFP and PARP1-Chr-RFP in HeLa cells. (**a**) Diffusion coefficient of untagged GFP in HeLa cells measured by the segmented FCS method. The mobility of GFP in the nucleolus is slower (D = 9.5 ± 3 µm^2^/s) compared to the nucleoplasm (D = 29 ± 4 µm^2^/s), due to the higher molecular crowding inside the nucleolar compartment. Data are shown as mean ± S.D. (**b**) Diffusion coefficient of PARP1-Chr-RFP in HeLa cells measured by the segmented FCS method. The diffusion coefficient of PARP1-Chr-RFP in HeLa cells is measured for the cytoplasmic (D = 4.5 ± 2.7 µm^2^/s), nucleoplasm (D = 1.3 ± 0.4 µm^2^/s), and nucleolar regions (D = 0.4 ± 0.2 µm^2^/s).

As a validation of our method, we measured the mobility of untagged GFP in the nucleus. As expected, the diffusion was slowed down in the nucleolus relative to the nucleoplasm (Fig. 5a), presumably because of the higher molecular crowding of the nucleolar compartment. We also showed the applicability of our method in the study of slower molecular diffusion, like in the case of PARP1 chromobody tagged with RFP (PARP1-Chr-RFP). Also in this case, we found that diffusion was slowed down in the nucleolus relative to the nucleoplasm (Fig. 5b) but more likely because of specific interaction of PARP1 with the large amount of RNA contained in the nucleolar region^37^. This result highlights once again the importance of accuracy in determining the subcellular region of interest when performing FCS measurement. Subcellular components can move during the acquisition of FCS data. For this reason, we rely on relative intensity variations to identify and select the segments corresponding to a specific subcellular region.

## Conclusions

In summary, we described a method to perform segmented FCS in commercial LSMs that are not equipped with FCS setups. The method can be used to perform accurate mobility measurements in solutions and live cells. We believe that this new tool will be useful to study the dynamics of molecules within chromatin, eventually leading to interesting insights into nuclear structure–function relationships.

### Supplementary Information

Supplementary Figures.

## Data Availability

Data sets generated during the current study are available from the corresponding author on reasonable request.
