# Supplementary material for: Segmented fluorescence correlation spectroscopy (FCS) on a commercial laser scanning microscope
Source: Sci Rep. 2024 Jul 30;14:17555. doi: 10.1038/s41598-024-68317-7 (PMC11289089; doi:10.1038/s41598-024-68317-7)
Supplement: Supplementary file 1 — Supplementary Figures. [file 41598_2024_68317_MOESM1_ESM.pdf]

Supporting Information for:

Segmented Fluorescence Correlation Spectroscopy (FCS) on a commercial laser scanning microscope

Elisa Longo[1], Silvia Scalisi[1], Luca Lanzanò [1,2]*

[1]Department of Physics and Astronomy "Ettore Majorana", University of Catania, Catania, Italy; [2]Nanoscopy, CHT Erzelli, Istituto Italiano di Tecnologia, Genoa, Italy;

*Corresponding author:

Luca Lanzanò
Department of Physics and Astronomy "Ettore Majorana", University of Catania
Via S. Sofia, 64 - 95123 Catania (Italy)
Email: luca.lanzano@unict.it
Phone: +39 095 3785330

This file contains Supplementary Figures S1-S2

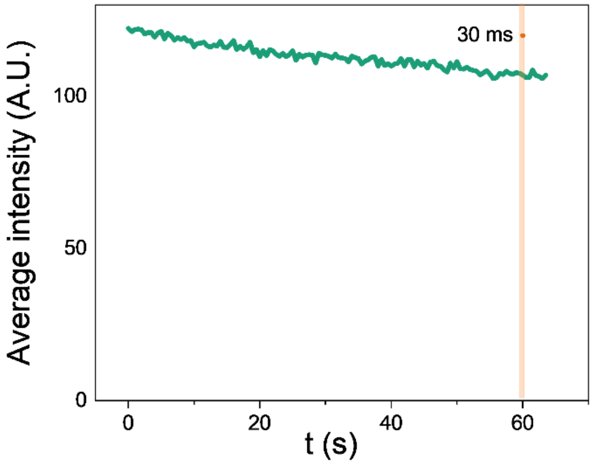

Fig. S1. Photobleaching during FCS of untagged GFP in HeLa cells. Representative intensity time profile for an acquisition of untagged GFP in the nucleus of HeLa cell. The duration of the segment used for the analysis (T=30 ms) is indicated for comparison.

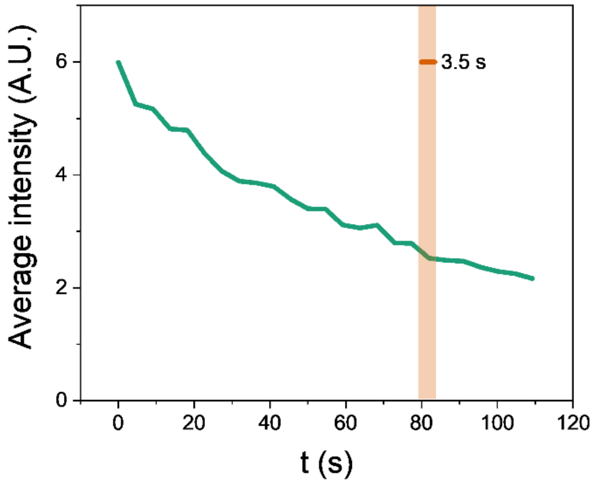

Fig. S2. Photobleaching during FCS of PARP1-chr-RFP in HeLa cells. Representative intensity time profile for an acquisition of PARP1-chr-RFP in the nucleus of HeLa cell. The duration of the segment used for the analysis (T=3.5 s) is indicated for comparison.
